# Interfacial modification in perovskite-based tandem solar cells

**DOI:** 10.1186/s40580-023-00374-6

**Published:** 2023-05-20

**Authors:** Ik Jae Park, Hyo Kyung An, Yuna Chang, Jin Young Kim

**Affiliations:** 1 Department of Materials Physics, Sookmyung Women’s University, Seoul, 04310 Republic of Korea; 2 Institute of Advanced Materials and Systems, Sookmyung Women’s University, Seoul, 04310 Republic of Korea; 3 Department of Materials Science and Engineering, Seoul National University, Seoul, 08826 Republic of Korea; 4 Research Institute of Advanced Materials, Seoul National University, Seoul, 08826 Republic of Korea

**Keywords:** Perovskites, Tandem, Interfaces, Solar cells

## Abstract

With photovoltaic performance of metal halide perovskite-based solar cells skyrocketing to approximately 26% and approaching the theoretical Shockley–Queisser limit of single junction solar cells, researchers are now exploring multi-junction tandem solar cells that use perovskite materials to achieve high efficiency next-generation photovoltaics. Various types of bottom subcells, including silicon solar cells used commercially in industry, chalcogenide thin film cells, and perovskite cells, have been combined with perovskite top subcells on the strength of facile fabrication methods based on solution processes. However, owing to the nature that photovoltages of the subcells are added up and the structure containing numerous layers, interfacial issues that cause open-circuit voltage (*V*_OC_) deficit need to be handled carefully. In addition, morphological issues or process compatibility make it difficult to fabricate solution-processed perovskite top cells. In this paper, we summarize and review the fundamentals and strategies to overcome interfacial issues in tandem solar cells for high efficiency and stability confronting this field.

**Graphical Abstract:**

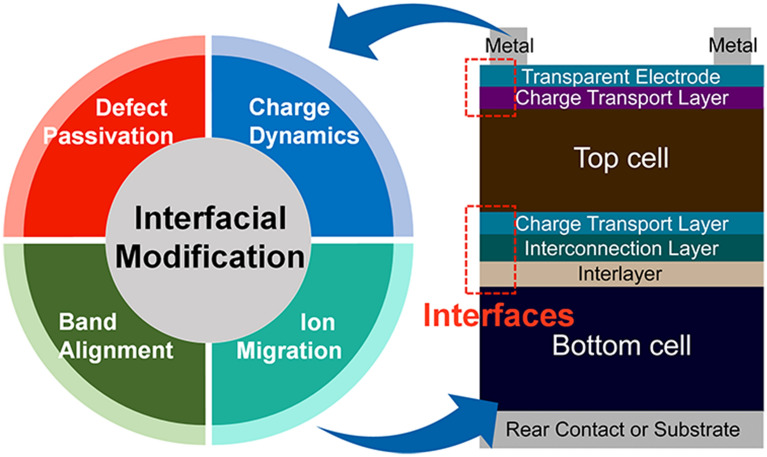

## Introduction

Since a solid-state perovskite solar cell (PSC) was reported to achieve a power conversion efficiency (PCE) of approximately 10%, [1–3] organic-inorganic metal halide perovskite materials have received tremendous attention as next-generation optoelectronic materials. Based on their excellent electrical and optical properties, as well as their facile solution process, which is applicable to flexible substrates, device performance has been rapidly increasing up to 25.8% for a decade [4]. This efficiency value is comparable to that of a single crystal silicon solar cell (26.1%) whose installed capacity is rapidly increasing for zero carbon emissions. It is also approaching theoretically feasible efficiency of single junction solar cells [5]. To overcome efficiency limit, tandem solar cells (TSCs) that connect two or more cells in series have been developed. It exploits more than two absorbers with different optical bandgaps to utilize incident sunlight efficiently by reducing thermalization and non-absorption losses. As the bandgap of perovskite can be easily tuned by compositional engineering, various perovskite-based TSCs with Si, Cu(In,Ga)Se_2_ (CIGS), Cu_2_ZnSn(S,Se)_4_ (CZTSSe), GaAs, and organic solar cells as bottom subcells have been studied [6–16]. Among them, the perovskite/Si tandem cell has achieved 33.2% efficiency, surpassing the theoretical limit of single junction solar cells [4].

One of the main challenges with TSCs is achieving high efficiency. Due to the numerous functional layers compared to the single junction cell, the probability of photogenerated carrier losses at the interface is high, thus interfacial modification at all interfaces should be a prerequisite to secure high efficiency as well as stability. Typical strategies demonstrated in single-junction PSCs have been adopted at electron transport layers (ETLs)/perovskite and hole transport layers (HTLs)/perovskite interfaces in TSCs, such as passivation and modifying electronic band structures. Additionally, the design of the recombination layer and top electrodes is also crucial.

In this review, we summarized basic concepts and types of TSCs and reviewed the recent progress on interfacial modification in perovskite-based TSCs. Interface engineering is one of the most important strategies to achieve high efficiency and stability. The discussion in this paper will provide research guidance for further development.

## Concept and structure of tandem solar cells

**Fig. 1:**
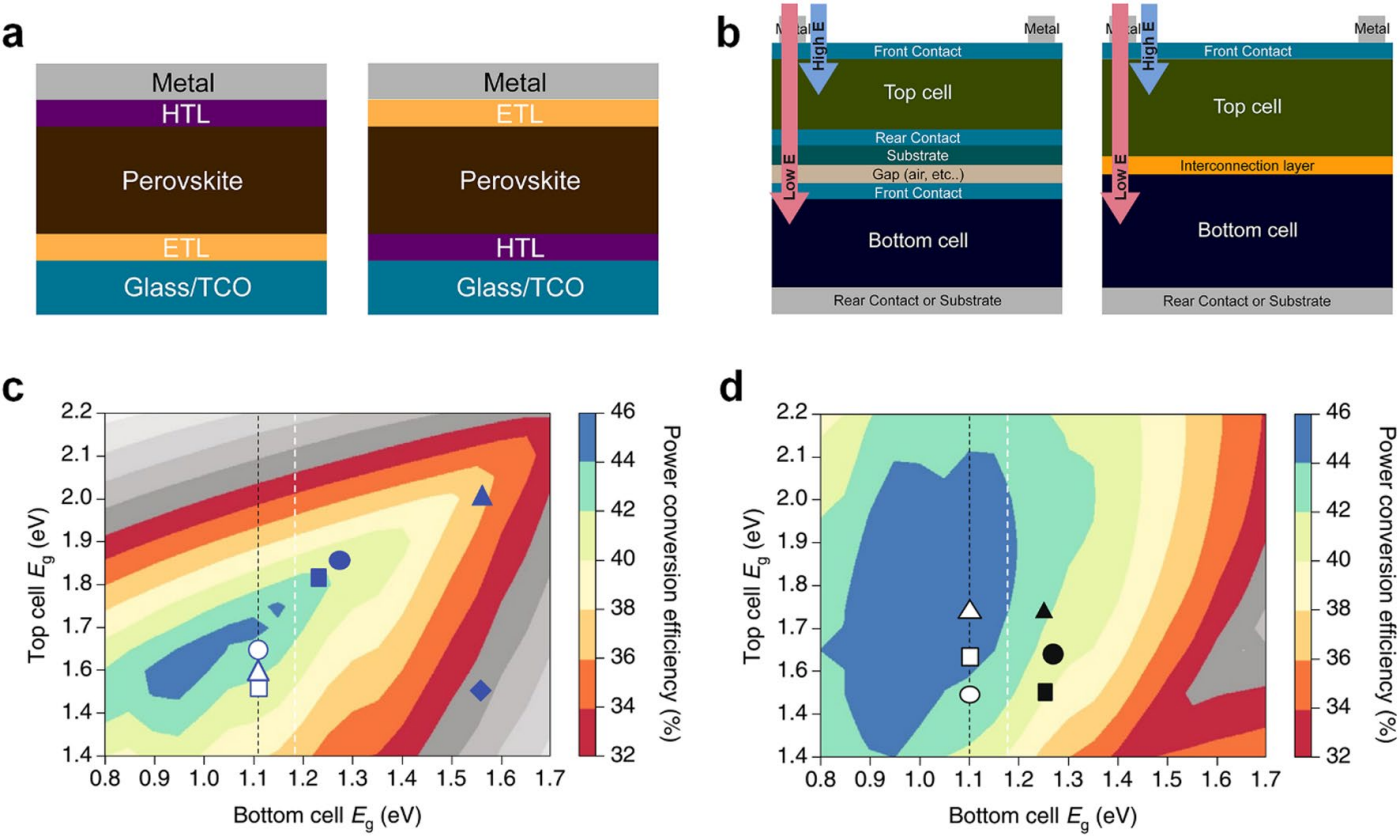
A schematic illustration of **a** the n-i-p (left), p-i-n (right) structure, and **b** mechanically stacked 4-T (left), monolithic 2-T (right) TSCs. Theoretical efficiency limit **c** for 2-T and **d** for 4-T tandems, calculated with different bandgap combination. The black dahsed line marks a bandgap of Si. The white dashed line marks the lowest bandgap of perovskite materials. Grey shading region indicates no efficiency gain by tandem cells. Reproduced with permission [79]. Copyright 2018, Springer Nature

The perovskite solar cells can be classified based on the direction of charge carrier collection into two types: n-i-p (normal) and p-i-n (inverted). It is designated by stacking order of the charge transport layer (CTL) and the perovskite from the substrate. Figure 1a shows planar n-i-p and p-i-n structure perovskite solar cells. Although, high efficiency n-i-p cells widely employ a nano-sized mesoporous oxide layer to facilitate charge extraction, [17–19] we focus on planar structure here for convenience. Both types of cells use different CTLs due to process compatibility. For example, TiO_2_ has been used as the ETL for the n-i-p type due to its excellent electrical/optical properties, however, it is not suitable for the p-i-n type due to limited compatibility. Typically, the n-i-p type cell employs TiO_2_ (or SnO_2_) as ETLs and 2,2ʹ,7,7ʹ-tetrakis(*N*,*N*-dimethoxyphenylamine)-9,9ʹ-spirobifluorene (spiro-MeOTAD) (or poly(triaryl amine) (PTAA)) as HTLs, respectively [20–22]. On the other hand, the p-i-n type cell uses thermally evaporated C_60_ (or [6]-phenyl C_61_-butyric acid methyl ester (PCBM)) as ETLs and nickel oxide (NiO), poly(3,4-ethylenedioxythiophene)-poly(styrenesulfonate) (PEDOT:PSS), PTAA as HTLs [23, 24]. For TSCs, the incident light should pass through into the upper side, composed of the transparent top electrode and the CTL. In this case, CTLs correspond to ETLs for p-i-n and HTLs for n-i-p, respectively. Top-side CTLs must have excellent optical properties and protect the perovskite layer from damage during the sputtering process for deposition of the transparent conducting oxide (TCO) layer. Because spiro-MeOTAD and PTAA layers significantly absorb the light of short wavelength and cannot protect the underlying perovskite layers from sputtering damage, [25] the p-i-n structure is favorable for high efficiency perovskite-based TSCs.

To achieve high efficiency solar cells, it is crucial to utilize a wide range of sunlight without losses. The photoactive layer can absorb incident photons of higher energy than its bandgap. Lower energy photons pass through the absorber, leading to an absorption loss. In addition, there is a thermalization loss, where hot carriers generated by photons with much higher energy than the bandgap are relaxed to the band edge as heat [26]. The TSC, which employs two or more absorber layers with different bandgap, is an effective way to mitigate these losses. The incident light enters toward a wide-bandgap absorber, i.e., the top subcell, and high energy photons are absorbed first. Unabsorbed low energy photons are captured by a narrow bandgap absorber layer in the bottom subcell. As a result, reduced absorption and thermalization losses by multi-absorption layers lead to a higher photon-to-power conversion efficiency compared to the single junction solar cell.

Multi-junction solar cells can utilize a wide range of incident light using a spectrum-splitting dichroic mirror or a tandem structure that stacks subcells vertically. Generally, there are two types of the tandem solar cell depending on the device structure: monolithic two-terminal (2-T) and mechanical four-terminal (4-T). As shown in Fig. 1b, the monolithic 2-T TSC consists of the two subcells directly connected by a recombination layer or a tunnel junction, which is fabricated on a single substrate. On the other hand, the mechanical 4-T TSC is composed of two subcells that are mechanically stacked after separate fabrication on each substrate. For the 2-T tandem cell, no additional electrodes or substrates, e.g., glasses, are needed because two subcells are electrically connected by a thin recombination layer (less than tens of nanometers scale) on a single substrate. Therefore, the 2-T TSC is more favorable than the 4-T TSC in terms of light harvesting. However, subcells are coupled by series connection, amounts of electrons and holes generated by top and bottom subcells have to be identical to operate efficiently. Thus, overall current of the tandem cell is limited by the lower current subcell. To maximize total current density of the tandem cell, those of subcells must be exactly matched using optical engineering. Figure 1c shows that the combination of the bandgap of subcells should be carefully considered because the efficiency is very sensitive to current matching.

In the 4-T TSC, the top subcell is placed on the bottom subcell, resulting in inevitable absorption and reflection losses by a transparent electrode, a substrate, and a gap between subcells. However, the top and bottom subcells are electrically decoupled from each other, eliminating the deed for current matching between subcells, making the combination of subcell bandgap less important [27]. Furthermore, the 4-T TSC has the advantage of a facile fabrication process. It is not necessary to consider any detrimental factors that could affect the bottom cell, e.g., dissolution by solvents, or damage by temperature. Despite these advantages, 2-T TSCs have received attention due to favorable light harvesting. The solar cell parameters of recently reported 2-T TSCs are summarized in Table 1.

**Table 1:** Solar cell parameters of reported perovskite-based TSCs.

Perovskite cell type	Tandem structure	*J*_SC_ (mA cm^–2^)	*V*_OC_ (V)	Fill Factor	PCE (%)	Area (cm^2^)	Refs.
p-i-n	CIGS/AZO/2PACz /PVSK/C_60_/SnO_2_/IZO/LiF	19.17	1.68	0.719	23.26	1.03	[59]
p-i-n	c-Si(flat)/ITO/Me-4PACz /PVSK/LiF/C_60_/SnO_2_/IZO/Ag/LiF	19.26	1.90	0.795	29.15	1.064	[13]
p-i-n	c-Si(textured)/ITO/MeO-2PACz/PVSK/C_60_/SnO_2_/IZO/Ag/NaF	18.57	1.69	0.789	24.72	1.008	[80]
p-i-n	c-Si(textured)/ITO/NiO_x_/N719/PVSK/LiF/C_60_/SnO_x_/IZO/Ag/MgF_2_	19.2	1.78	0.768	26.2	1.03	[61]
p-i-n	c-Si(textured)/ITO/NiO_x_/2PACz/PVSK/LiF/C_60_/SnO_2_/IZO/Ag/LiF	20.11	1.794	0.799	28.84	1.2	[62]
n-i-p	c-Si(flat)/ITO/c-TiO_2_/m-TiO_2_/PCBM:PMMA/PVSK/spiro-MeOTAD/ITO/Au/MgF_2_	15.2	1.837	0.773	21.6	0.25	[81]
p-i-n	ITO/PTAA/PVSK/C_60_/SnO_1.76_/PVSK/C_60_/BCP/Cu	15.2 (15.1)	2.03 (2.01)	0.797 (0.732)	24.6 (22.2)	0.059 (1.15)	[69]
p-i-n	c-Si(flat)/ITO/PTAA/PVSK/C_60_/SnO_2_/IZO/LM foil	18.5	1.76	0.785	25.5	0.77	[75]
p-i-n	CIGS/BZO/ITO/PTAA/PVSK/PCBM/ZnO NPs/ITO/MgF_2_	17.3	1.774	0.731	22.43	0.042	[12]
p-i-n	CZTSSe/ITO/PTAA/PVSK/C_60_/ITO/Ag/LiF	17.67	1.460	0.68	17.5	0.4	[11]
p-i-n	c-Si(flat)/ITO/NiO/PVSK/LiF/PCBM/SnO_2_/ZTO/ITO/Ag/LiF	18.1	1.65	0.79	23.6	1	[8]
p-i-n	c-Si(textured)/IZO/2PACz/PVSK/MgF_x_/C_60_/SnO_2_/IZO/Ag/MgF_x_	19.80	1.91	0.776	29.30	1	[14]
p-i-n	c-Si(textured)/nc-Si:H(n+)/nc-Si:H(p+)/spiro-TTB/PVSK/LiF/C_60_/SnO_2_/IZO/Ag/MgF_2_	19.53	1.79	0.731	25.52	1.42	[64]
p-i-n	c-Si(flat)/nc-SiO_x_:H/ITO/polyTPD/PVSK/ETL/ITO/AR	19.02	1.79	0.746	25.43	1.1	[66]
p-i-n	ITO/PTAA/PVSK/C_60_/SnO_2_/Au/PEDOT:PSS/PVSK/C_60_/BCP/Cu	15.6 (14.0)	1.97 (1.95)	0.81 (0.82)	24.8 (22.3)	0.073 (1.05)	[67]
p-i-n	c-Si(textured)/ITO/NiO/SAMs/PVSK/C_60_/SnO_2_/IZO/Ag/MgF_2_	19.8 (18.5)	1.85 (1.79)	0.789 (0.757)	28.9 (25.1)	1.05 (16)	[82]
p-i-n	ITO/NiO/VNPB/PVSK/C_60_/SnO_2_/ITO NCs/PVSK/C_60_/SnO_2_/Cu	16.2	2.03	0.803	26.3	0.049	[68]
p-i-n	ITO/NiO_x_/PVSK/C_60_/BCP/Ag NPs/MoO_x_/PBDBT-2 F:Y6:PC_71_BM/TPBi/Ag	13.05	1.902	0.831	20.60	0.062	[72]
p-i-n	ITO/MeO-2PACz/PVSK/PC_61_BM/SnO_x_/InO_x_/MoO_x_/PM6/Y6/C_60_/BCP/Ag	14.0	2.15	0.80	24.0	0.0174	[10]
p-i-n	ITO/NiO_x_/BPA/PVSK/C_60_/BCP/IZO/MoO_x_/OPV/PNDIT-F3N/Ag	14.83 (14.24)	2.06 (2.06)	0.772 (0.744)	23.60 (21.77)	0.08 (1.05)	[73]
p-i-n	c-Si(flat)/ITO/PTAA/PVSK/C_60_/PEIE/ITO/Ag/MgF_2_	19.53	1.87	0.776	28.37	1	[7]
p-i-n	c-Si(flat)/ITO/PTAA/PVSK/C_60_/PEIE/ITO/Ag/Motheye	19.2	1.756	0.792	26.7	1	[6]
p-i-n	c-Si(textured)/ITO/PTAA/PVSK/C_60_/SnO_2_/ITO/Ag/PDMS	19.2	1.82	75.3	26.2	0.4225	[74]
p-i-n	c-Si(textured)/ITO/PTAA/LiF/PVSK/C_60_/SnO_2_/ITO/Ag/PDMS	19	1.92	78.5	28.6	1	[15]

## Interface engineering in perovskite-based tandem cells

The concept of interface engineering in perovskite-based tandem cells is similar to that of single-junction perovskite solar cells. The goal is to collect photogenerated carriers to electrodes without any losses during transfer and transport. One of the most influential factors that determine the efficiency of the solar cell is charge recombination, which can be classified into three types: Shockley-Read-Hall (SRH) recombination, radiative recombination, and Auger recombination [28]. SRH and Auger recombinations are sorted as non-radiative recombination. Non-radiative recombination induces an open-circuit voltage (*V*_OC_) loss that limits efficiencies of solar cells. SRH recombination is called as trap-assisted recombination because it is related to traps, and SRH recombination is the dominant non-radiative recombination process. Thus, SRH recombination can be accelerated by defects in bulk materials or at the interfaces. It significantly affects the photovoltaic performance of TSCs in that TSCs consist of numerous interfaces. Therefore, minimizing non-radiative charge recombination by interface engineering is crucial to achieving highly efficient and stable TSCs. We will introduce several functions of interface engineering in PSCs. The roles of interface engineering are defect passivation, improving charge-carrier dynamics, band alignment, suppressing ion migration, and these are closely related to each other. Later, we will discuss research on interface engineering in the TSC below.

**Fig. 2:**
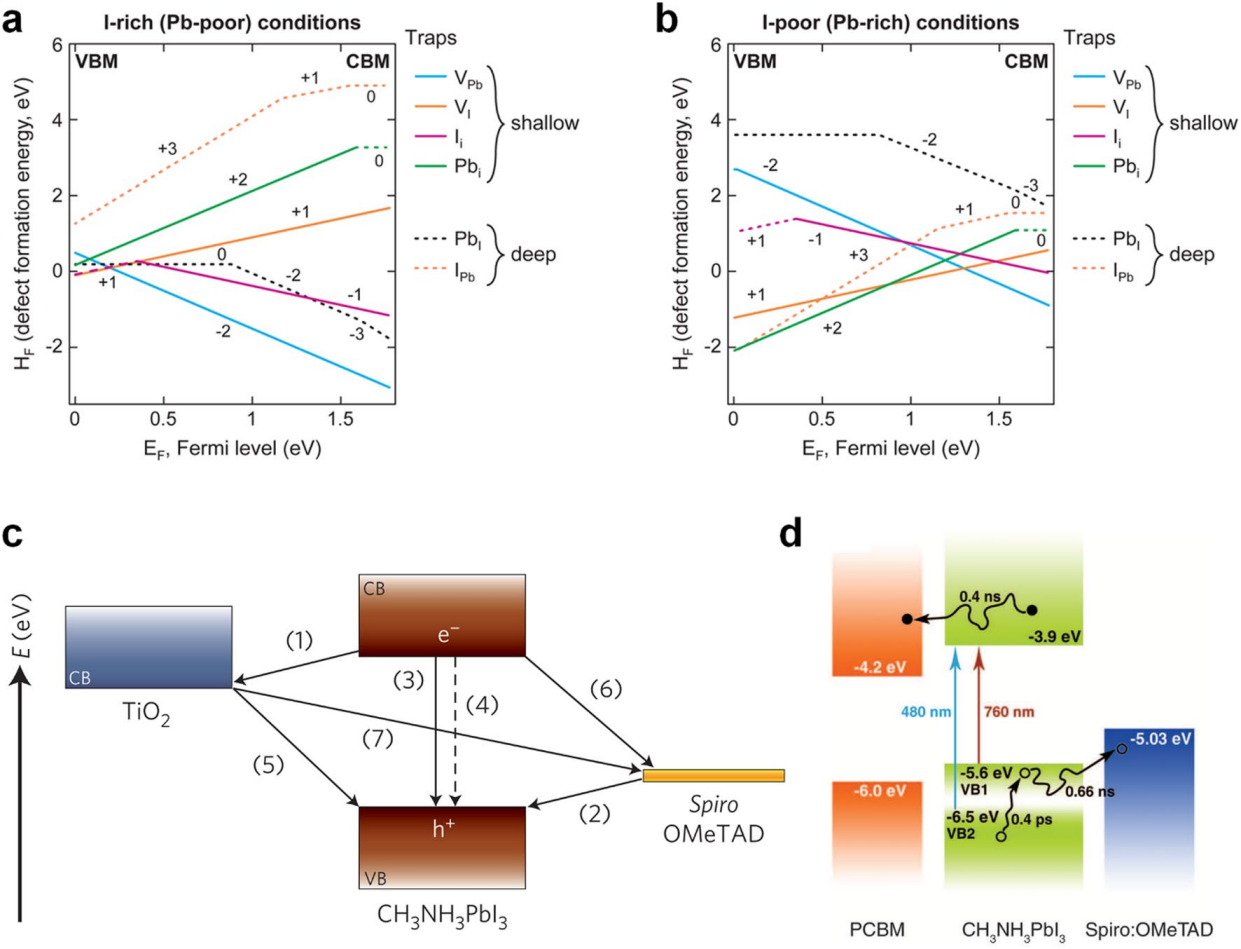
Defect formation energies for tetragonal MAPbI_3_**a** under halide rich and **b** under halide poor conditions. Reproduced with permission [30]. Copyright 2015, American Chemical Society. **c** Schematic diagram of energy levels and electron transfer processes in a spiro-MeOTAD/perovskite/TiO_2_ cell. Reproduced with permission [38]. Copyright 2014, Springer Nature. **d** Schematic diagram of the hot-hole cooling, charge recombination in MAPbI_3_, and charge separation at interfaces. Reproduced with permission [37]. Copyright 2013, American Association for the Advancement of Science

*Defect Passivation* Defects in perovskite bulk materials or at surfaces are generated due to the low temperature process, which leads to shallow and deep-level traps in the energy level. Figure 2a and b show that the perovskite materials contain intrinsic point defects, including vacancies (V_Pb_, V_X_), interstitials (Pb_i_, X_i_), and antisite substitutions (Pb_X_, X_Pb_) [29, 30]. While vacancies and interstitials dominantly exhibit shallow transition energy levels, antisite defects induce deep-level traps which are more detrimental defects than shallow traps [31]. Among them, a Pb_I_ antisite defect has quite low defect formation energy at the surface from the calculation by density functional theory (DFT) [32]. The perovskite material was known as defect tolerant, [33, 34] however, photogenerated carriers can be recombined by deep-level traps or accumulated at the interfaces, leading to *V*_OC_ losses and the hysteresis effect. Besides the surface of the perovskite material, there are several interfaces, e.g., interconnecting layers (ICLs)/CTLs and CTLs/electrodes, which exist the detrimental defects, thus all interfaces should be carefully designed to achieve high performance devices.

*Improving Charge-Carrier Dynamics* Charge-carrier dynamics are related to a series of processes involving extraction, transfer, transport, and recombination of photogenerated charge carriers in materials and at surfaces. When a perovskite layer absorbs incident light, excitons are generated in the material. These photogenerated excitons should be separated into free charge carriers. Fortunately, metal halide perovskite materials have a favorable exciton binding energy of 14–25 meV, which is smaller than the thermal energy at room temperature (~ 26 meV) [35, 36]. Free electrons and holes by dissociation of excitons are extracted to CTLs at the interfaces within the timescale of picoseconds as shown in Fig. 2c and d [37, 38]. High quality perovskite materials have lifetimes of several microseconds, which is enough time to extract charge carriers at the interfaces before they recombine [39–41]. For efficient charge collection, the formation of the built-in electric field at the perovskite/CTL interface and enhanced conductivity of the CTL are crucial. Seok and coworkers demonstrated the tailoring Zn_2_SnO_4_ quantum dot (QD) ETL by controlling particle size, leading to a larger built-in field [42]. In addition, doping of NiO layers with metals such as Cu or Li has been studied to enhance charge transport properties in p-i-n type PSCs [43, 44].

*Band Alignment* Energy level band alignment is deeply correlated with the aforementioned charge dynamics. Adjustment of the band level alignment is crucial to minimize *V*_OC_ losses. Photogenerated electrons and holes have to be separated to opposite direction: perovskite/HTL and perovskite/ETL, respectively. Thus, the highest occupied molecular orbital (HOMO) level (or valence band maximum, VBM) of HTLs and VBM of perovskite absorbers, and the lowest unoccupied molecular orbital (LUMO) level (or conduction band minimum, CBM) of ETLs and CBM of perovskite should be matched for holes and electrons transfer, respectively. For electron injection and hole extraction, driving forces within 0.2 eV are theoretically appropriate [45]. In p-i-n type devices, i.e., most of the TSCs, low work function metals such as Ag and Cu are used for facilitating charge transfer from the ETL to electrodes. Au metal electrode has too large a work function to transfer electrons from ETLs to electrode.

**Fig. 3:**
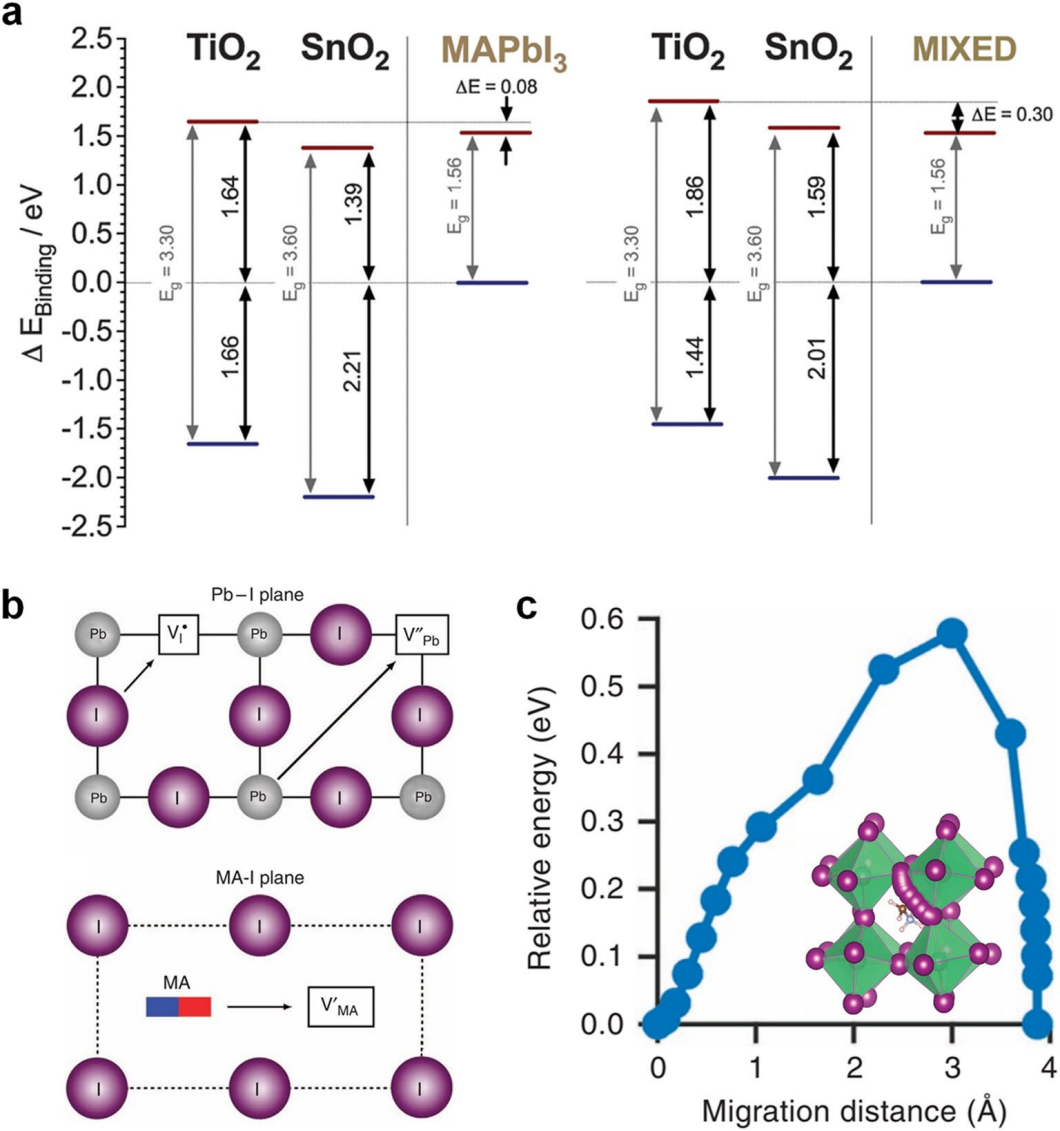
**a** Energy level diagrams of TiO_2_ and SnO_2_-based PSCs for MAPbI_3_ (left) and (FAPbI_3_)_0.85_(MAPbBr_3_)_0.15_ (right). Reproduced with permission [47]. Copyright 2015, The Royal Society of Chemistry. **b** Schematic illustration of the ion migration mechanisms involving conventional vacancy hopping between neighbouring position. **c** Calculated energy profile depending on the migration distance. Inset shows migration path. Reproduced with permission [49]. Copyright 2015, Springer Nature

For an example of band alignment modification, Zhou and coworkers demonstrated that ultrathin (1 to 10 nm) polyethyleneimine ethoxylated (PEIE) polymer surface modifier can reduce the work function (WF) of the conductors due to the intrinsic molecular dipole moments associated with the neutral amine groups and the charge transfer character of the interaction with the conductor [46]. This work suggested a universal method to form low WF contacts. In Fig. 3a, Baena and coworkers showed that SnO_2_ ETL deposited by atomic layer deposition (ALD) improves the conduction band misalignment of TiO_2_ by a favorable alignment of the conduction band, mitigating the energy barrier [47]. They achieved hysteresis-free planar PSCs with high voltages.

*Suppressing Ion Migration* Perovskite materials have an ionic conduction property. Some of cations (MA^+^, Pb^2+^) and anions (I^–^, Br^–^) in perovskite absorbers can easily migrate in thin films (Fig. 3b and c). The halide perovskite material has a low activation energy of ion migration that makes ion diffusion easy under electrical bias or light illumination during the operation conditions. It induces a hysteresis effect depending on the sweep direction, or light soaking effect [48–51]. Generally, halide anion migration adversely affects device performance and stability in PSCs. For example, I^–^ anions move towards metal contact of Ag or Cu. These ions chemically react and convert to AgI or CuI layers, which hinder charge transfer at the interface [52–54].

For TSCs, a 1.68–1.7 eV or wider bandgap perovskite absorber has been used for perovskite-based TSCs, and perovskite contains a high content of Br over 20 mol%. Highly Br-contained compositions are vulnerable to phase segregation into I- and Br-rich region in bulk materials under light illumination [55–57]. The most effective way to exclude phase segregation in I-Br mixed halide composition is to exploit a pure halide wide bandgap composition, [7] however the Br content of most of wide bandgap perovskite is still high. Since the formation of defects at the surface dominates ion migration, interface engineering is useful to mitigate the negative effects of ion migration [58].

### Perovskite/CTL Interfaces

**Fig. 4:**
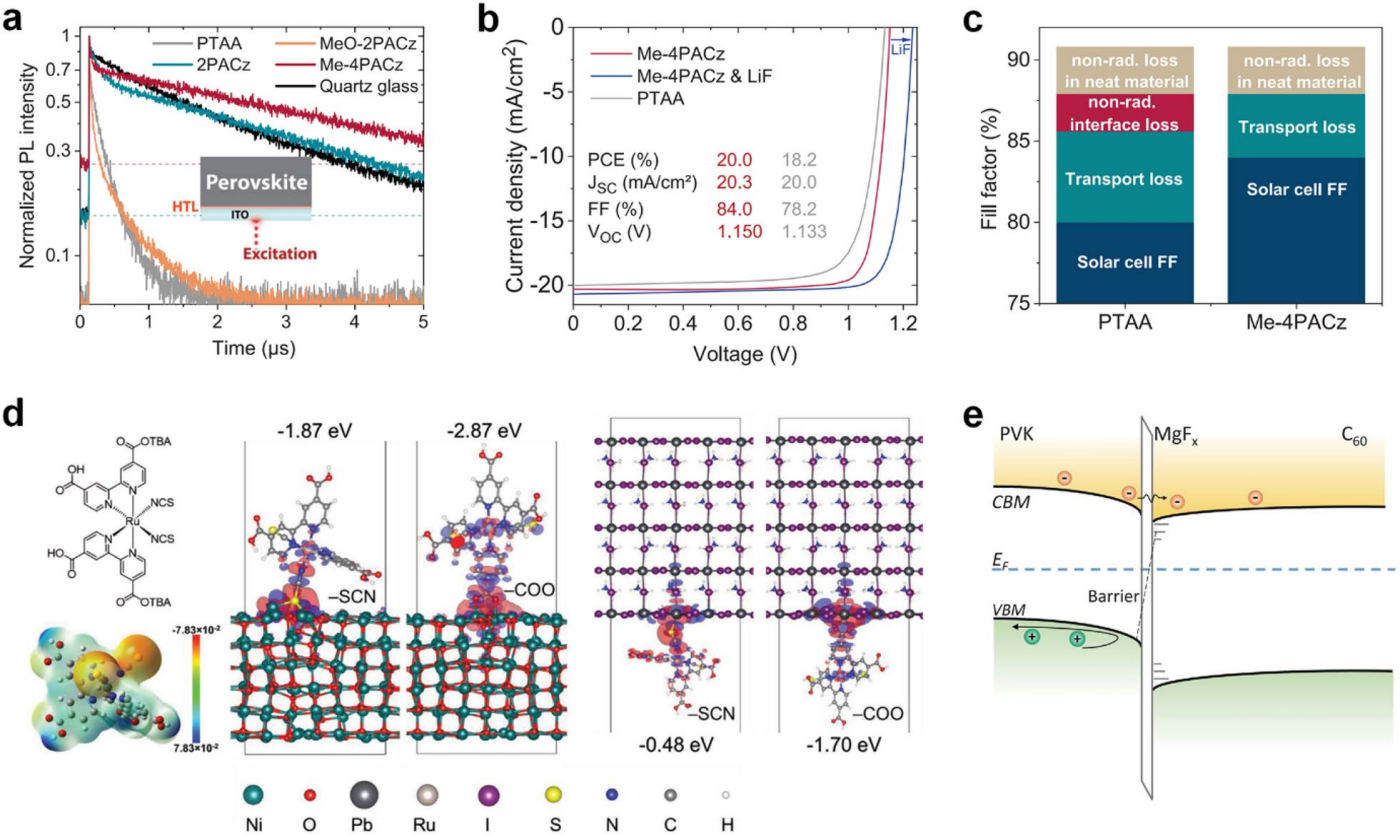
**a** PL transients of ITO/HTL/perovskite. The dashed lines marks the background levels. **b** Current density–voltage (*J*–*V*) characteristics of the best single junction PSCs composed of glass/ITO/HTL/perovskite/C_60_/SnO_2_/Ag. c Repartition of the fill factor (FF) loss mechanism of PSCs with PTAA and Me-4PACz. Reproduced with permission [13]. Copyright 2020, American Association for the Advancement of Science. **d** Molecular structure (upper left), electrostatic potential surface of the N719 (lower left), charge density differences for NiO(001)/N719 (middle) and N719/PbI_2_-rich MAPbI_3_(001) (right) interfaces through –SCN and –COO binding modes using DFT calculation (at GGA/PBE + vdW level of theory). Reproduced with permission [61]. Copyright 2020, Wiley-VCH. **e** Schematic diagram of energy levels of the perovskite/MgF_x_/C_60_ interfaces. Reproduced with permission [14]. Copyright 2022, American Association for the Advancement of Science

Interface engineering has been investigated in TSCs at perovskite/CTLs interfaces. Albrecht and coworkers introduced molecules based on carbazole with phosphonic acid groups, MeO-2PACz ([2-(3,6-dimethoxy-9 H-carbazol-9-yl)ethyl]phosphonic acid) and 2PACz ([2-(9 H-carbazol-9-yl)ethyl]phosphonic acid). It can form self-assembled monolayers (SAMs) conformally on recombination layers, even on rough surfaces of bottom cells such as CIGS cells [59]. Later, Albrecht and coworkers modified SAMs with a methyl group substitution, named Me-4PACz ([4-(3,6-dimethyl-9 H-carbazol-9-yl)butyl]phosphonic acid), for perovskite/Si tandem cells as shown in Fig. 4a and c [13]. They demonstrated that the key issue for high efficiency is to lower the ideality factor while minimizing non-radiative recombination. Me-4PACz-based perovskite/Si TSCs exhibited a certified PCE of 29.15%, and the initial PCE was retained at 95% after 300 h under maximum power point tracking (MPPT) in ambient air without encapsulation.

For inorganic materials, nickel oxide has been widely used as an HTL for PSCs due to its chemical stability, cheap price, suitable VBM, excellent optical and electrical properties, as well as high versatility [24, 44, 60]. Wolf and coworkers focused on the passivation of defects at the NiO_x_/perovskite interface originated from Ni^≥ 3+^ states due to a nickel deficiency at the NiO_x_ surfaces (Fig. 4d) [61]. The metal organic dye molecule (N719), widely used in dye-sensitized solar cells, provides passivation effects on both the sputtered NiO_x_ layer and perovskite surfaces, leading to improved charge carrier dynamics and a PCE of 26.2%, as well as excellent thermal stability at 85 °C. Liu and coworkers exploited NiO_x_/2PACz ultrathin double layers to form conformal HTL onto textured Si bottom cells and obtained a certified PCE of 28.84% by minimizing shunt losses [62].

For perovskite/ETL interfaces, Wolf and coworkers investigated metal fluorides (NaF, CaF_x_, LiF, MgF_x_) as the interlayer by thermal evaporation at the perovskite/ETL (C_60_) interface (Fig. 4e) [14]. Among them, a ~ 1 nm thick MgF_x_ layer adjusted the surface energy of the perovskite and displaced C_60_ from the perovskite surfaces, facilitating electron extraction from the perovskite to C_60_ and suppressing interface recombination. The perovskite/Si TSCs with MgF_x_ exhibited a high *V*_OC_ of 1.92 V, resulting in a PCE of 29.3% and high stability.

Similar strategies using cysteine hydrochloride (CysHCl) were suggested by Zhao and coworkers. CysHCl acted as a bulky passivator and a surface anchoring agent. Perovskite/C_60_ interfaces treated by CysHCl showed reduced trap density and suppressed non-radiative recombination. Vacuum level (*E*_vac_) and Fermi level (*E*_F_) shift toward CBM also occurred after treatment, resulting in the energy band bending downward at the interfaces. It induced facilitated electron transfer from perovskite to C_60_ and blocked the holes at the interfaces [63].

Huang and coworkers introduced a reducing agent benzylhydrazine hydrochloride (BHC) to prevent Sn^2+^ oxidation in narrow bandgap Sn–Pb perovskites for a hot gas-assisted blading method. BHC-contained perovskites exhibited improved carrier recombination lifetime and enabled laser scribing in ambient conditions after air exposure for a few minutes. It resulted from the formation of a thin SnO_2_ layer between perovskite/ETL interfaces during air exposure. As a result, BHC boosted the efficiency of the all-perovskite tandem mini module to 21.6% (an aperture area of 14.3 cm^2^) with superior photostability [64].

Huang and coworkers also showed gradient doping in narrow bandgap Sn–Pb perovskites by Ba^2+^ ions to modify bulk perovskites and interfaces for all-perovskite tandem cells. BaI_2_-contained perovskite precursors enabled heterogeneous distribution of Ba^2+^ ions in perovskite films. It is found that Ba^2+^ ions can turn the top region of the perovskite film to n-type without changing the bandgap. Drive-level capacitance profiling (DLCP) measurements confirmed reduced doping levels near perovskite/C_60_ interfaces. The gradient doping led to a built-in field in the films, facilitating charge extraction [65].

### Interconnecting layers

In 2-T TSCs, there are two types of ICLs where photogenerated eletrons and holes are recombined to maintain charge neutrality between subcells: A tunnel junction and a metallic recombination layer. Tunnel junctions are generally composed of highly doped n- and p-type layers, while a metallic recombination layer uses a single transparent conductive layer such as an indium tin oxide (ITO) layer. To achieve highly efficient TSCs, ICLs should be designed carefully. It requires three functions: (1) excellent optical properties, (2) efficient charge carrier recombination, and (3) protection of the bottom cell during the top cell process.

**Fig. 5:**
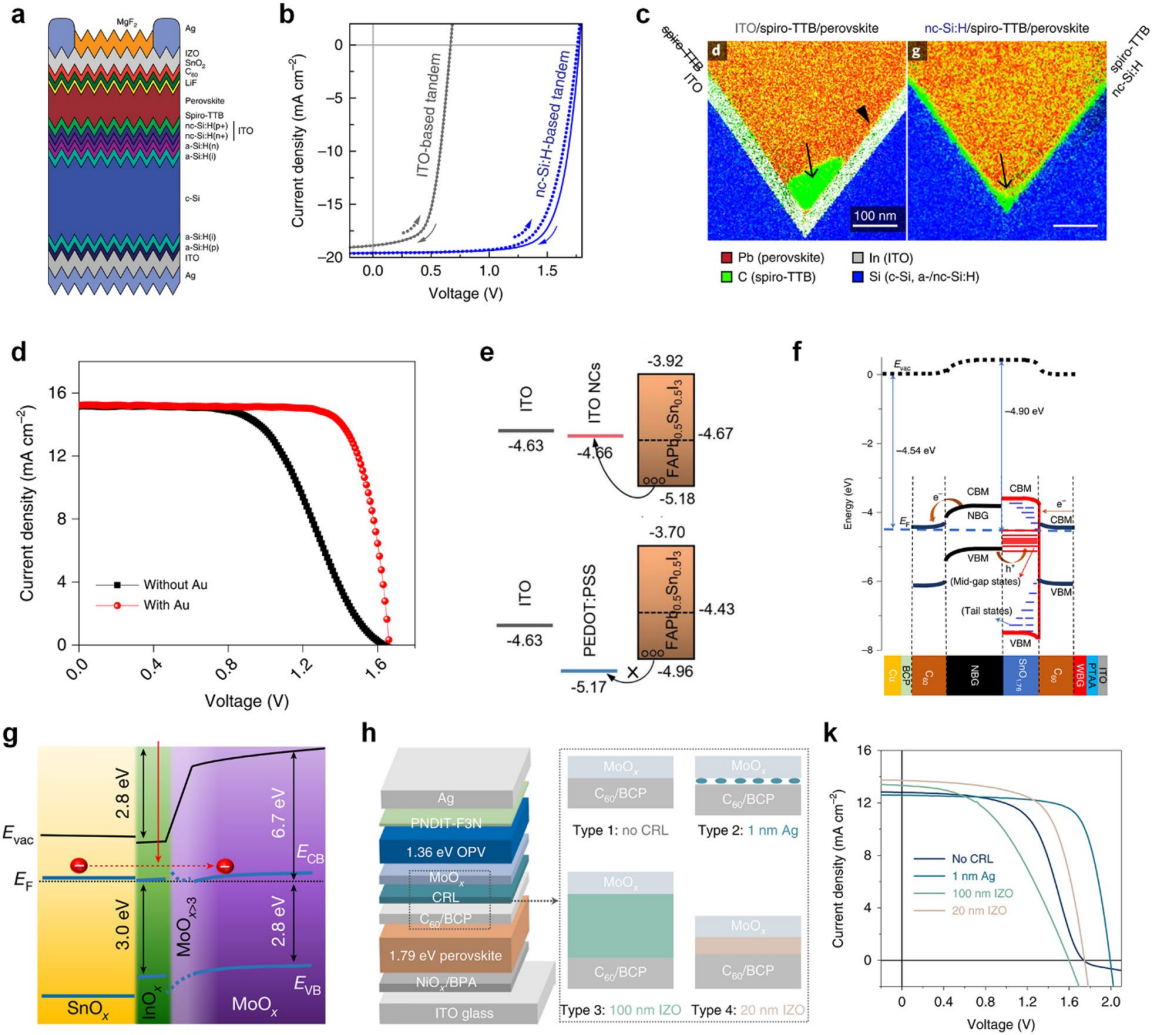
**a** Schematic illustration of a fully textured perovskite/Si TSCs. **b ***J*–*V* characteristics of TSCs with ITO/spiro-TTB and nc-Si:H/spiro-TTB interfaces described in Fig. 5a. **c** STEM EDX map of a cross-section of ITO/spiro-TTB and nc-Si:H/spiro-TTB interfaces. Reproduced with permission [67]. Copyright 2018, Springer Nature. **d ***J*–*V* characteristics of TSCs with and without an ultrathin Au layer in the tunnel junction. Reproduced with permission [70]. Copyright 2019, Springer Nature. **e** Energy level diagram of the all-FA perovskite on ITO/PEDOT:PSS and ITO/ITO NCs. Reproduced with permission [71]. Copyright 2022, Wiley-VCH. **f** Energy level diagram (top) for the C_60_/SnO_1.76_/perovskite(narrow *E*_g_)/C_60_ layers in all-perovskite TSCs (device structure at bottom). Reproduced with permission.[72] Copyright 2020, Springer Nature. **g** Energy level diagram of the SnO_x_/InO_x_/MoO_x_ layers for perovskite/organic TSCs. Reproduced with permission [10]. Copyright 2022, Springer Nature. **h** Schematic diagram and **k ***J*–*V* characteristics of the perovskite/organic TSCs with various ICLs. Reproduced with permission [76]. Copyright 2022, Springer Nature

In Fig. 5a and c, Sahli and coworkers proposed a tunnel junction with n- and p-type nanocrystalline hydrogenerated silicon (nc-Si:H(n+) and nc-Si:H(p+)) for perovskite/Si TSCs [66, 67]. Using a tunnel junction, optical losses and shunt resistance can be reduced, leading to the increase of the bottom cell photocurrent by more than 1 mA cm^–2^. They also achieved fully textured 2-T perovskite/Si TSCs with a 2,2ʹ,7,7ʹ-tetra(N,N-di-tolyl)amino-9,9-spiro-bifluorene (spiro-TTB) and a tunnel junction. Conformally deposited spiro-TTB by thermal evaporation was accumulated at the valley of Si cells during annealing process when they used ITO/spiro-TTB, which caused the top cell short. On the other hand, spiro-TTB was fully covered on nc-Si:H even after annealing. Ho-Baillie and coworkers demonstrated perovskite/Si TSCs using a tunnel junction of SnO_2_/p + + with a homo-junction silicon cell [68]. It enabled n-i-p type perovskite/Si TSCs with large areas of 4 and 16 cm^2^.

Snaith and coworkers proposed a light management strategy using nanocrystalline silicon oxide [69]. They adopted a 110 nm thick interlayer with a refractive index of 2.6 (at a wavelength of 800 nm) instead of amorphous hydrogenerated silicon or nc-Si:H. After optimization, the current density of the Si bottom cell was improved by 1.4 mA cm^–2^.

In case of perovskite/perovskite TSCs, ICLs have a crucial function of preventing the penetration of the perovskite solution into the bottom cell during the top cell process besides the charge recombination. Various combinations of layers have been studied to design effective ICLs with excellent optical properties. Tan and coworkers exploited a ~ 1 nm thick Au layer on C_60_/ALD-SnO_2_ layers (Fig. 5d) [70]. The ALD-SnO_2_ layer provided improved electron extraction prevented damage to the underlying cells. An Au film facilitated charge carrier recombination, replacing the TCO layer. Later, researchers achieved higher efficiency using ITO nanocrystals (NCs) (Fig. 5e) [71]. The band level of All-FA narrow bandgap perovskite and ITO NCs is well matched compared to the conventional PEDOT:PSS, enabling a stabilized PCE of 26.3% TSCs with high thermal stability. In Fig. 5f, Huang and coworkers demonstrated simple ICLs composed of C_60_/SnO_1.76_ without any metals or TCO layers [72]. The fullerene and SnO_1.76_ layer formed an ohmic contact due to the unintentional n-doping of C_60_ by iodine anions from perovskite. The SnO_1.76_ layer exhibited an ambipolar carrier transport property by the presence of a high density of Sn^2+^, enabling simplified ICLs without TCO layers.

Organic solar cells generally use non-polar solvents, such as chloroform for precursor solutions. As a result, the fabrication of perovskite/organic TSCs is relatively favorable compared to perovskite/perovskite TSCs. Since non-fullerene acceptors have been introduced, the efficiency of organic solar cells has dramatically increased, [73, 74] thus perovskite/organic TSCs have received attention. Various ICLs have been employed for perovskite/organic TSCs. Yang and coworkers introduced Ag nanoparticles (NPs) as an ICL with negligible optical loss and high reproducibility [75]. Recently, Riedl and coworkers demonstrated that an ultrathin ALD-InO_x_ interlayer between SnO_x_ and MoO_x_ CTLs eliminated energy barriers and facilitated charge recombination without optical losses as shown in Fig. 5g [10]. Hou and coworkers achieved a PCE of 23.6% perovskite/organic TSC using a 4 nm thick sputtered indium zinc oxide ICL (Fig. 5 h and k) [76].

### Others

**Fig. 6:**
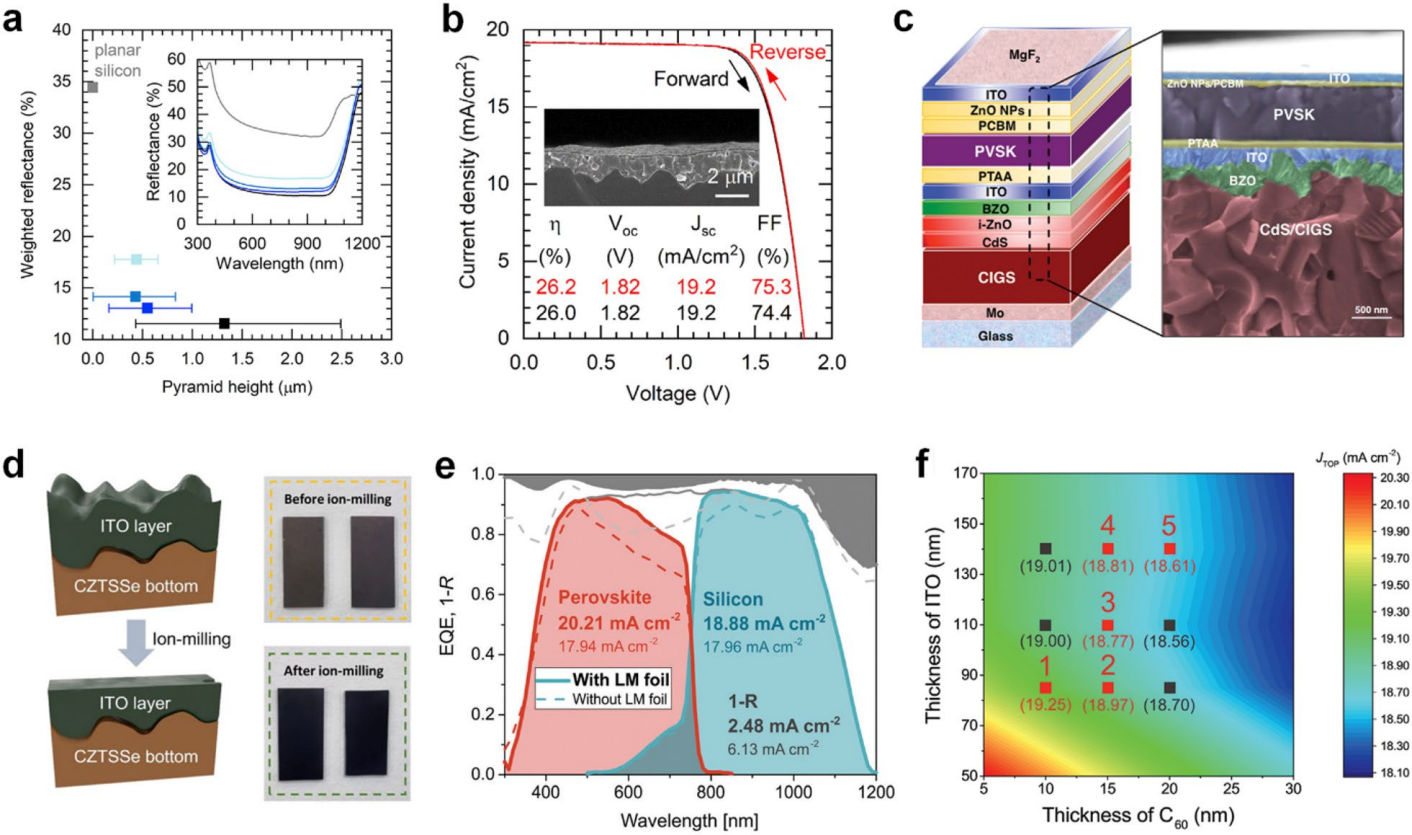
**a** AM1.5G-weighted (700–1000 nm) reflectance with various pyramid height. The inset shows the reflectance spectra. **b ***J*–*V* characteristics of the PDMS/Planarized TSCs depending on the scan direction. Reproduced with permission [77]. Copyright 2020, Elsevier. **c** Schematic illustration and SEM image of the 2-T perovskite/CIGS TSC. Reproduced with permission [12]. Copyright 2018, American Association for the Advancement of Science. **d** A schematic illustration and photos of CZTSSe bottom cells before and after ion-milling of ITO layers. Reproduced with permission [11]. Copyright 2022, John Wiley and Sons. **e** External quantum efficiency (EQE) and 1–*R* (reflectance) spectra of the TSCs with and without LM foil. Reproduced with permission [78]. Copyright 2018, The Royal Society of Chemistry. **f** Contour map of the theoretically calculated *J*_SC_ of the perovskite top cell depending on the thickness of the ITO and C_60_ layers. Reproduced with permission [25]. Copyright 2021, Wiley-VCH.

In this section, we will review the morphology control of bottom cells and interfacial modification at the top electrode. Most of perovskite layers are deposited using solution processes, e.g., spin-coating and blade coating, onto bottom cells. However, due to the < 1 μm thickness of perovskite top cells, it is quite tricky to form perovskite top cells conformally on textured Si cells or chalcogenide thin film cells with bumpy surfaces. Figure 6a and b show that Huang and coworkers fabricated a solution-based blading process for perovskite-based TSCs onto textured silicon bottom cells [77]. The reduced pyramid height of the front surface of the Si cell enabled the formation of the blade-coated perovskite top cells, minimizing reflectance caused by textured bottom cells. An optimized dimethyl sulfoxide (DMSO)/Pb ratio in the precursor solution led to void-free and fully covered perovskite films. The perovskite layer can be formed at a speed of 1.5 m min^–1^, which corresponds to more than one wafer per second. They also exploited a polydimethylsiloxane (PDMS) light-scattering layer and achieved a PCE of 26.2% perovskite/Si TSC.

For chalcogenide thin film solar cells, surface flattening of the bottom cell is an effective strategy for morphology control. In Fig. 6c, Yang and coworkers proposed polished ICLs composed of i-ZnO, boron-doped ZnO, and ITO chemically and mechanically to deposit heavily doped PTAA HTLs [12]. Flattened bottom cells enabled uniform deposition of perovskite top cells, resulting in a 22.43% efficiency perovskite/CIGS tandem cell. Kim and coworkers also demonstrated reducing surface roughness of the CZTSSe bottom cell using the potentiostatic mode and the ion-milling process (Fig. 6d) [11]. The CZT precursor and CZTSSe films prepared by the potentiostatic method exhibited significantly reduced surface roughness (*R*_rms_). The top surface of a CdS/ITO layer was polished by the ion-milling, reducing *R*_rms_ from 107.67 to 22.39 nm. As a result, the perovskite/CZTSSe tandem cell exhibited a PCE of 17.5%.

For efficient light harvesting, the top electrode must have excellent optical and electrical properties. Additionally, it is essential to protect a perovskite layer from sputtering damage during the deposition of the top electrode. Albrecht and coworkers modified the top electrode to minimize the reflectance of incident light at the top surfaces by employing a light management (LM) foil as shown in Fig. 6e [78]. Improved short-circuit current density (*J*_SC_) due to reduced optical losses lead to an increase in efficiency from 23.4 to 25.5%. Kim and coworkers simulated the current density of the perovskite top cell depending on the thickness of ITO and C_60_ layers (Fig. 6f) [25]. They demonstrated a trade-off between the conductivity and the transparency of ITO. Additionally, the thickness of the C_60_ layer was optimized because a layer that is too thin can’t protect the perovskite from sputtering damage.

## Conclusions and future outlook

In summary, we reviewed interfacial modification for high efficiency and stable perovskite-based TSCs. Most of highly efficient perovskite-based TSCs employed a p-i-n structure to improve light harvesting and protect the perovskite layer from sputtering damage. Monolithic 2-T TSCs can harvest more incident light and are cheaper compared to 4-T TSCs. However, the fabrication process of the 2-T TSC is tricky due to single substrate and current matching between subcells. As the optical bandgap of perovskite can be highly tunable by compositional engineering, various TSCs combined with silicon, CIGS, CZTSSe, organic, and perovskite cells have been developed.

For 2-T monolithic tandem cells, interface engineering is the most effective way to improve photovoltaic performance. Interface engineering plays a key role in (1) defect passivation, (2) improving charge-carrier dynamics, (3) band alignment, and (4) suppressing ion migration at the interfaces. It leads to effective photogenerated charge collection to the electrodes without non-radiative charge carrier recombination by traps or charge accumulation at the interfaces as well as improved stability. For I-Br mixed halide composition, interfacial modification mitigated halide segregation by suppressing ion migration. Interfacial modification also improves light harvesting and enables coating of uniform layers on the rough surface of the bottom cell by morpholoy control. Various functional materials employed at the interfaces provided minimizing *V*_OC_ deficit as well as achieving high efficiency and stability.

Future research will focus on the development of multifunctional materials for interface engineering. Advanced materials must simultaneously meet low price, process compatibility, scalability, and chemical stability. It will allow us to achieve large scale, low-cost, and highly efficient multi-junction solar cells in photovoltaic industry.

## Data Availability

The datasets used and/or analysed during the current study are available from the corresponding author on reasonable request.

## References

[1] Kim H, Lee C, Im J, Lee K, Moehl T, Marchioro A, Moon S, Humphry-Baker R, Yum J, Moser JE, Grätzel M, Park N (2012). Lead iodide perovskite sensitized all-solid-state submicron thin film mesoscopic solar cell with efficiency exceeding 9%. Sci. Rep..

[2] Lee MM, Teuscher J, Miyasaka T, Murakami TN, Snaith HJ (2012). Efficient hybrid solar cells based on meso-superstructured organometal halide perovskites. Science.

[3] Kojima A, Teshima K, Shirai Y, Miyasaka T (2009). Organometal halide perovskites as visible-light sensitizers for photovoltaic cells. J. Am. Chem. Soc..

[4] Best research-cell efficiencies: http://www.Nrel.Gov (Accessed: April 2023)

[5] Shockley W, Queisser HJ (1961). Detailed balance limit of efficiency of p-n junction solar cells. J. Appl. Phys..

[6] Kim D, Jung HJ, Park IJ, Larson BW, Dunfield SP, Xiao C, Kim J, Tong J, Boonmongkolras P, Ji SG, Zhang F, Pae SR, Kim M, Kang SB, Dravid V, Berry JJ, Kim JY, Zhu K, Kim DH, Shin B (2020). Efficient, stable silicon tandem cells enabled by anion-engineered wide-bandgap perovskites. Science.

[7] Ji SG, Park IJ, Chang H, Park JH, Hong GP, Choi BK, Jang JH, Choi YJ, Lim HW, Ahn YJ, Park SJ, Nam KT, Hyeon T, Park J, Kim DH, Kim JY (2022). Stable pure-iodide wide-band-gap perovskites for efficient Si tandem cells via kinetically controlled phase evolution. Joule.

[8] Bush KA, Palmstrom AF, Yu ZJ, Boccard M, Cheacharoen R, Mailoa JP, McMeekin DP, Hoye RLZ, Bailie CD, Leijtens T, Peters IM, Minichetti MC, Rolston N, Prasanna R, Sofia S, Harwood D, Ma W, Moghadam F, Snaith HJ, Buonassisi T, Holman ZC (2017). Bent, MD McGehee, 23.6%-efficient monolithic perovskite/silicon tandem solar cells with improved stability. Nat. Energy.

[9] Tong J, Jiang Q, Ferguson AJ, Palmstrom AF, Wang X, Hao J, Dunfield SP, Louks AE, Harvey SP, Li C, Lu H, France RM, Johnson SA, Zhang F, Yang M, Geisz JF, McGehee MD, Beard MC, Yan Y, Kuciauskas D, Berry JJ, Zhu K (2022). Carrier control in Sn–Pb perovskites via 2D cation engineering for all-perovskite tandem solar cells with improved efficiency and stability. Nat. Energy.

[10] Brinkmann KO, Becker T, Zimmermann F, Kreusel C, Gahlmann T, Theisen M, Haeger T, Olthof S, Tückmantel C, Günster M, Maschwitz T, Göbelsmann F, Koch C, Hertel D, Caprioglio P, Peña-Camargo F, Perdigón-Toro L, Al-Ashouri A, Merten L, Hinderhofer A, Gomell L, Zhang S, Schreiber F, Albrecht S, Meerholz K, Neher D, Stolterfoht M, Riedl T (2022). Perovskite–organic tandem solar cells with indium oxide interconnect. Nature.

[11] S.K. Hwang, I.J. Park, S.W. Seo, J.H. Park, S.J. Park, J.Y. Kim, Electrochemically deposited CZTSSe thin films for monolithic perovskite tandem solar cells with efficiencies over 17%. Energy Environ. Mater. 0, e12489 (2022)

[12] Han Q, Hsieh Y, Meng L, Wu J, Sun P, Yao E, Chang S, Bae S, Kato T, Bermudez V, Yang Y (2018). High-performance perovskite/Cu(in,Ga)Se_2_ monolithic tandem solar cells. Science.

[13] Al-Ashouri A, Köhnen E, Li B, Magomedov A, Hempel H, Caprioglio P, Márquez JA, Morales Vilches AB, Kasparavicius E, Smith JA, Phung N, Menzel D, Grischek M, Kegelmann L, Skroblin D, Gollwitzer C, Malinauskas T, Jošt M, Matič G, Rech B, Schlatmann R, Topič M, Korte L, Abate A, Stannowski B, Neher D, Stolterfoht M, Unold T, Getautis V, Albrecht S (2020). Monolithic perovskite/silicon tandem solar cell with > 29% efficiency by enhanced hole extraction. Science.

[14] Liu J, De Bastiani M, Aydin E, Harrison GT, Gao Y, Pradhan RR, Eswaran MK, Mandal M, Yan W, Seitkhan A, Babics M, Subbiah AS, Ugur E, Xu F, Xu L, Wang M, Rehman A, Razzaq A, Kang J, Azmi R, Said AA, Isikgor FH, Allen TG, Andrienko D, Schwingenschlögl U, Laquai F, De Wolf S (2022). Efficient and stable perovskite-silicon tandem solar cells through contact displacement by MgF_x_. Science.

[15] Yang G, Ni Z, Yu ZJ, Larson BW, Yu Z, Chen B, Alasfour A, Xiao X, Luther JM, Holman ZC, Huang J (2022). Defect engineering in wide-bandgap perovskites for efficient perovskite–silicon tandem solar cells. Nat. Photonics.

[16] Li Z, Kim TH, Han SY, Yun Y-J, Jeong S, Jo B, Ok SA, Yim W, Lee SH, Kim K, Moon S, Park J-Y, Ahn TK, Shin H, Lee J, Park HJ (2020). Wide-bandgap perovskite/gallium arsenide tandem solar cells. Adv. Energy Mater..

[17] Jeong J, Kim M, Seo J, Lu H, Ahlawat P, Mishra A, Yang Y, Hope MA, Eickemeyer FT, Kim M, Yoon YJ, Choi IW, Darwich BP, Choi SJ, Jo Y, Lee JH, Walker B, Zakeeruddin SM, Emsley L, Rothlisberger U, Hagfeldt A, Kim DS, Grätzel M, Kim JY (2021). Pseudo-halide anion engineering for α-FAPbI_3_ perovskite solar cells. Nature.

[18] Zhang T, Wang F, Kim H, Choi I, Wang C, Cho E, Konefal R, Puttisong Y, Terado K, Kobera L, Chen M, Yang M, Bai S, Yang B, Suo J, Yang S, Liu X, Fu F, Yoshida H, Chen WM, Brus J, Coropceanu V, Hagfeldt A, Brédas J, Fahlman M, Kim DS, Hu Z, Gao F (2022). Ion-modulated radical doping of spiro-OMeTAD for more efficient and stable perovskite solar cells. Science.

[19] Kim G, Min H, Lee KS, Lee DY, Yoon SM, Seok SI (2020). Impact of strain relaxation on performance of α-formamidinium lead iodide perovskite solar cells. Science.

[20] Kim M, Jeong J, Lu H, Lee TK, Eickemeyer FT, Liu Y, Choi IW, Choi SJ, Jo Y, Kim H, Mo S, Kim Y, Lee H, An NG, Cho S, Tress WR, Zakeeruddin SM, Hagfeldt A, Kim JY, Grätzel M, Kim DS (2022). Conformal quantum dot–SnO_2_ layers as electron transporters for efficient perovskite solar cells. Science.

[21] Park J, Kim J, Yun H, Paik MJ, Noh E, Mun HJ, Kim MG, Shin TJ, Seok SI (2023). Controlled growth of perovskite layers with volatile alkylammonium chlorides. Nature.

[22] Yang WS, Park B, Jung EH, Jeon NJ, Kim YC, Lee DU, Shin SS, Seo J, Kim EK, Noh JH, Seok SI (2017). Iodide management in formamidinium-lead-halide–based perovskite layers for efficient solar cells. Science.

[23] Hu S, Otsuka K, Murdey R, Nakamura T, Truong MA, Yamada T, Handa T, Matsuda K, Nakano K, Sato A, Marumoto K, Tajima K, Kanemitsu Y, Wakamiya A (2022). Optimized carrier extraction at interfaces for 23.6% efficient tin–lead perovskite solar cells. Energy Environ. Sci..

[24] Park IJ, Kang G, Park MA, Kim JS, Seo SW, Kim DH, Zhu K, Park T, Kim JY (2017). Highly efficient and uniform 1cm^2^ perovskite solar cells with an electrochemically deposited NiO_x_ hole-extraction layer. Chem. Sus. Chem..

[25] Park IJ, Kim DH, Ji SG, Ahn YJ, Park SJ, Kim D, Shin B, Kim JY (2021). Rationally designed window layers for high efficiency perovskite/Si tandem solar cells. Adv. Opt. Mater.

[26] Shah J (1978). Hot electrons and phonons under high intensity photoexcitation of semiconductors. Solid State Electron.

[27] Lee J, Hsieh Y, De Marco N, Bae S, Han Q, Yang Y (2017). Halide perovskites for tandem solar cells. J. Phys. Chem. Lett..

[28] Sarritzu V, Sestu N, Marongiu D, Chang X, Masi S, Rizzo A, Colella S, Quochi F, Saba M, Mura A, Bongiovanni G (2017). Optical determination of Shockley-Read-Hall and interface recombination currents in hybrid perovskites. Sci. Rep..

[29] Kim JY, Lee J, Jung HS, Shin H, Park N (2020). High-efficiency perovskite solar cells. Chem. Rev..

[30] Buin A, Comin R, Xu J, Ip AH, Sargent EH (2015). Halide-dependent electronic structure of organolead perovskite materials. Chem. Mater..

[31] Tan H, Jain A, Voznyy O, Lan X, García de Arquer FP, Fan JZ, Quintero-Bermudez R, Yuan M, Zhang B, Zhao Y, Fan F, Li P, Quan LN, Zhao Y, Lu Z, Yang Z, Hoogland S, Sargent EH (2017). Efficient and stable solution-processed planar perovskite solar cells via contact passivation. Science.

[32] Wang R, Xue J, Wang K, Wang Z, Luo Y, Fenning D, Xu G, Nuryyeva S, Huang T, Zhao Y, Yang JL, Zhu J, Wang M, Tan S, Yavuz I, Houk KN, Yang Y (2019). Constructive molecular configurations for surface-defect passivation of perovskite photovoltaics. Science.

[33] Brandt RE, Poindexter JR, Gorai P, Kurchin RC, Hoye RLZ, Nienhaus L, Wilson MWB, Polizzotti JA, Sereika R, Žaltauskas R, Lee LC, MacManus-Driscoll J, Bawendi M, Stevanović V, Buonassisi T (2017). Searching for “defect-tolerant” photovoltaic materials: combined theoretical and experimental screening. Chem. Mater..

[34] Yin W, Shi T, Yan Y (2014). Unusual defect physics in CH_3_NH_3_PbI_3_ perovskite solar cell absorber. Appl. Phys. Lett..

[35] Miyata A, Mitioglu A, Plochocka P, Portugall O, Wang JT, Stranks SD, Snaith HJ, Nicholas RJ (2015). Direct measurement of the exciton binding energy and effective masses for charge carriers in organic–inorganic tri-halide perovskites. Nat. Phys..

[36] Galkowski K, Mitioglu A, Miyata A, Plochocka P, Portugall O, Eperon GE, Wang JT, Stergiopoulos T, Stranks SD, Snaith HJ, Nicholas RJ (2016). Determination of the exciton binding energy and effective masses for methylammonium and formamidinium lead tri-halide perovskite semiconductors. Energy Environ. Sci..

[37] Xing G, Mathews N, Sun S, Lim SS, Lam YM, Grätzel M, Mhaisalkar S, Sum TC (2013). Long-range balanced electron- and hole-transport lengths in organic-inorganic CH_3_NH_3_PbI_3_. Science.

[38] Marchioro A, Teuscher J, Friedrich D, Kunst M, van de Krol R, Moehl T, Grätzel M, Moser J (2014). Unravelling the mechanism of photoinduced charge transfer processes in lead iodide perovskite solar cells. Nat. Photonics.

[39] Tong J, Song Z, Kim DH, Chen X, Chen C, Palmstrom AF, Ndione PF, Reese MO, Dunfield SP, Reid OG, Liu J, Zhang F, Harvey SP, Li Z, Christensen ST, Teeter G, Zhao D, Al-Jassim M, van Hest, Maikel FAM, Beard MC, Shaheen SE, Berry JJ, Yan Y, Zhu K (2019). Carrier lifetimes of > 1 µs in Sn-Pb perovskites enable efficient all-perovskite tandem solar cells. Science.

[40] Han Q, Bai Y, Liu J, Du K, Li T, Ji D, Zhou Y, Cao C, Shin D, Ding J, Franklin AD, Glass JT, Hu J, Therien MJ, Liu J, Mitzi DB (2017). Additive engineering for high-performance room-temperature-processed perovskite absorbers with micron-size grains and microsecond-range carrier lifetimes. Energy Environ. Sci..

[41] Hutter EM, Sutton RJ, Chandrashekar S, Abdi-Jalebi M, Stranks SD, Snaith HJ, Savenije TJ (2017). Vapour-deposited cesium lead iodide perovskites: microsecond charge carrier lifetimes and enhanced photovoltaic performance. ACS Energy Lett..

[42] Shin SS, Yang WS, Yeom EJ, Lee SJ, Jeon NJ, Joo Y, Park IJ, Noh JH, Seok SI (2016). Tailoring of electron-collecting oxide nanoparticulate layer for flexible perovskite solar cells. J. Phys. Chem. Lett..

[43] Park M, Park IJ, Park S, Kim J, Jo W, Son HJ, Kim JY (2018). Enhanced electrical properties of Li–doped NiO_x_ hole extraction layer in p–i–n type perovskite solar cells. Curr. Appl. Phys..

[44] Kim JH, Liang P, Williams ST, Cho N, Chueh C, Glaz MS, Ginger DS, Jen AK (2015). High-performance and environmentally stable planar heterojunction perovskite solar cells based on a solution-processed copper-doped nickel oxide hole-transporting layer. Adv. Mater..

[45] Park N (2013). Organometal perovskite light absorbers toward a 20% efficiency low-cost solid-state mesoscopic solar cell. J. Phys. Chem. Lett..

[46] Zhou Y, Fuentes-Hernandez C, Shim J, Meyer J, Giordano AJ, Li H, Winget P, Papadopoulos T, Cheun H, Kim J, Fenoll M, Dindar A, Haske W, Najafabadi E, Khan TM, Sojoudi H, Barlow S, Graham S, Brédas J, Marder SR, Kahn A, Kippelen B (2012). A universal method to produce low–work function electrodes for organic electronics. Science.

[47] Baena JPC, Steier L, Tress W, Saliba M, Neutzner S, Matsui T, Giordano F, Jacobsson TJ, Srimath Kandada AR, Zakeeruddin SM, Petrozza A, Abate A, Nazeeruddin MK, Grätzel M, Hagfeldt A (2015). Highly efficient planar perovskite solar cells through band alignment engineering. Energy Environ. Sci..

[48] Calado P, Telford AM, Bryant D, Li X, Nelson J, O’Regan BC, Barnes PRF (2016). Evidence for ion migration in hybrid perovskite solar cells with minimal hysteresis. Nat. Commun..

[49] Eames C, Frost JM, Barnes PRF, O’Regan BC, Walsh A, Islam MS (2015). Ionic transport in hybrid lead iodide perovskite solar cells. Nat. Commun..

[50] Zhang T, Cheung SH, Meng X, Zhu L, Bai Y, Ho CHY, Xiao S, Xue Q, So SK, Yang S (2017). Pinning down the anomalous light soaking effect toward high-performance and fast-response perovskite solar cells: the ion-migration-induced charge accumulation. J. Phys. Chem. Lett..

[51] Liu J, Hu M, Dai Z, Que W, Padture NP, Zhou Y (2021). Correlations between electrochemical ion migration and anomalous device behaviors in perovskite solar cells. ACS Energy Lett..

[52] Kato Y, Ono LK, Lee MV, Wang S, Raga SR, Qi Y (2015). Silver iodide formation in methyl ammonium lead iodide perovskite solar cells with silver top electrodes. Adv. Mater. Interfaces.

[53] Besleaga C, Abramiuc LE, Stancu V, Tomulescu AG, Sima M, Trinca L, Plugaru N, Pintilie L, Nemnes GA, Iliescu M, Svavarsson HG, Manolescu A, Pintilie I (2016). Iodine migration and degradation of perovskite solar cells enhanced by metallic electrodes. J. Phys. Chem. Lett..

[54] Li X, Fu S, Zhang W, Ke S, Song W, Fang J (2020). Chemical anti-corrosion strategy for stable inverted perovskite solar cells. Sci. Adv..

[55] Anaya M, Lozano G, Calvo ME, Míguez H (2017). ABX_3_ perovskites for tandem solar cells. Joule.

[56] McMeekin DP, Sadoughi G, Rehman W, Eperon GE, Saliba M, Hörantner MT, Haghighirad A, Sakai N, Korte L, Rech B, Johnston MB, Herz LM, Snaith HJ (2016). A mixed-cation lead mixed-halide perovskite absorber for tandem solar cells. Science.

[57] Hoke ET, Slotcavage DJ, Dohner ER, Bowring AR, Karunadasa HI, McGehee MD (2015). Reversible photo-induced trap formation in mixed-halide hybrid perovskites for photovoltaics. Chem. Sci..

[58] Meggiolaro D, Mosconi E, De Angelis F (2019). Formation of surface defects dominates ion migration in lead-halide perovskites. ACS Energy Lett..

[59] Al-Ashouri A, Magomedov A, Roß M, Jošt M, Talaikis M, Chistiakova G, Bertram T, Márquez JA, Köhnen E, Kasparavičius E, Levcenco S, Gil-Escrig L, Hages CJ, Schlatmann R, Rech B, Malinauskas T, Unold T, Kaufmann CA, Korte L, Niaura G, Getautis V, Albrecht S (2019). Conformal monolayer contacts with lossless interfaces for perovskite single junction and monolithic tandem solar cells. Energy Environ. Sci..

[60] Park JH, Seo J, Park S, Shin SS, Kim YC, Jeon NJ, Shin H, Ahn TK, Noh JH, Yoon SC, Hwang CS, Seok SI (2015). Efficient CH_3_NH_3_PbI_3_ perovskite solar cells employing nanostructured p-type NiO electrode formed by a pulsed laser deposition. Adv. Mater..

[61] Zhumagali S, Isikgor FH, Maity P, Yin J, Ugur E, De Bastiani M, Subbiah AS, Mirabelli AJ, Azmi R, Harrison GT, Troughton J, Aydin E, Liu J, Allen T, Rehman A, Baran D, Mohammed OF, De Wolf S (2021). Linked nickel oxide/perovskite interface passivation for high-performance textured monolithic tandem solar cells. Adv. Energy Mater..

[62] Mao L, Yang T, Zhang H, Shi J, Hu Y, Zeng P, Li F, Gong J, Fang X, Sun Y, Liu X, Du J, Han A, Zhang L, Liu W, Meng F, Cui X, Liu Z, Liu M (2022). Fully textured, production-line compatible monolithic perovskite/silicon tandem solar cells approaching 29% efficiency. Adv. Mater..

[63] J. Luo, R. He, H. Lai, C. Chen, J. Zhu, Y. Xu, F. Yao, T. Ma, Y. Luo, Z. Yi, Y. Jiang, Z. Gao, J. Wang, W. Wang, H. Huang, Y. Wang, S. Ren, Q. Lin, C. Wang, F. Fu, D. Zhao, Improved carrier management via a multifunctional modifier for high-quality low-bandgap Sn–Pb perovskites and efficient all-perovskite tandem solar cells. Adv. Mater. 2300352 (2023) 10.1002/adma.202300352 36906929

[64] Dai X, Chen S, Jiao H, Zhao L, Wang K, Ni Z, Yu Z, Chen B, Gao Y, Huang J (2021). Efficient monolithic all-perovskite tandem solar modules with small cell-to-module derate. Nat. Energy.

[65] Yu Z, Chen X, Harvey SP, Ni Z, Chen B, Chen S, Yao C, Xiao X, Xu S, Yang G, Yan Y, Berry JJ, Beard MC, Huang J (2021). Gradient doping in Sn–Pb perovskites by barium ions for efficient single-junction and tandem solar cells. Adv. Mater..

[66] Sahli F, Kamino BA, Werner J, Bräuninger M, Paviet-Salomon B, Barraud L, Monnard R, Seif JP, Tomasi A, Jeangros Q, Hessler-Wyser A, De Wolf S, Despeisse M, Nicolay S, Niesen B, Ballif C (2018). Improved optics in monolithic perovskite/silicon tandem solar cells with a nanocrystalline silicon recombination junction. Adv. Energy Mater..

[67] Sahli F, Werner J, Kamino BA, Bräuninger M, Monnard R, Paviet-Salomon B, Barraud L, Ding L, Diaz Leon JJ, Sacchetto D, Cattaneo G, Despeisse M, Boccard M, Nicolay S, Jeangros Q, Niesen B, Ballif C (2018). Fully textured monolithic perovskite/silicon tandem solar cells with 25.2% power conversion efficiency. Nat. Mater..

[68] Zheng J, Lau CFJ, Mehrvarz H, Ma F, Jiang Y, Deng X, Soeriyadi A, Kim J, Zhang M, Hu L, Cui X, Lee DS, Bing J, Cho Y, Chen C, Green MA, Huang S, Ho-Baillie A (2018). Large area efficient interface layer free monolithic perovskite/homo-junction-silicon tandem solar cell with over 20% efficiency. Energy Environ. Sci..

[69] Mazzarella L, Lin Y, Kirner S, Morales-Vilches A, Korte L, Albrecht S, Crossland E, Stannowski B, Case C, Snaith HJ, Schlatmann R (2019). Infrared light management using a nanocrystalline silicon oxide interlayer in monolithic perovskite/silicon heterojunction tandem solar cells with efficiency above 25%. Adv. Energy Mater..

[70] Lin R, Xiao K, Qin Z, Han Q, Zhang C, Wei M, Saidaminov MI, Gao Y, Xu J, Xiao M, Li A, Zhu J, Sargent EH, Tan H (2019). Monolithic all-perovskite tandem solar cells with 24.8% efficiency exploiting comproportionation to suppress sn(II) oxidation in precursor ink. Nat. Energy.

[71] Wu P, Wen J, Wang Y, Liu Z, Lin R, Li H, Luo H, Tan H (2022). Efficient and thermally stable all-perovskite tandem solar cells using all-FA narrow-bandgap perovskite and metal-oxide-based tunnel junction. Adv. Energy Mater..

[72] Yu Z, Yang Z, Ni Z, Shao Y, Chen B, Lin Y, Wei H, Yu ZJ, Holman Z, Huang J (2020). Simplified interconnection structure based on C_60_/SnO_2-x_ for all-perovskite tandem solar cells. Nat. Energy.

[73] Yan C, Barlow S, Wang Z, Yan H, Jen AK, Marder SR, Zhan X (2018). Non-fullerene acceptors for organic solar cells. Nat. Rev. Mater.

[74] Cheng P, Li G, Zhan X, Yang Y (2018). Next-generation organic photovoltaics based on non-fullerene acceptors. Nat. Photonics.

[75] Chen X, Jia Z, Chen Z, Jiang T, Bai L, Tao F, Chen J, Chen X, Liu T, Xu X, Yang C, Shen W, Sha WEI, Zhu H, Yang Y (2020). Efficient and reproducible monolithic perovskite/organic tandem solar cells with low-loss interconnecting layers. Joule.

[76] Chen W, Zhu Y, Xiu J, Chen G, Liang H, Liu S, Xue H, Birgersson E, Ho JW, Qin X, Lin J, Ma R, Liu T, He Y, Ng AM, Guo X, He Z, Yan H, Djurišić AB, Hou Y (2022). Monolithic perovskite/organic tandem solar cells with 23.6% efficiency enabled by reduced voltage losses and optimized interconnecting layer. Nat. Energy.

[77] Chen B, Yu ZJ, Manzoor S, Wang S, Weigand W, Yu Z, Yang G, Ni Z, Dai X, Holman ZC, Huang J (2020). Blade-coated perovskites on textured silicon for 26%-efficient monolithic perovskite/silicon tandem solar cells. Joule.

[78] Jošt M, Köhnen E, Morales-Vilches A, Lipovšek B, Jäger K, Macco B, Al-Ashouri A, Krč J, Korte L, Rech B, Schlatmann R, Topič M, Stannowski B, Albrecht S (2018). Textured interfaces in monolithic perovskite/silicon tandem solar cells: Advanced light management for improved efficiency and energy yield. Energy Environ. Sci..

[79] Leijtens T, Bush KA, Prasanna R, McGehee MD (2018). Opportunities and challenges for tandem solar cells using metal halide perovskite semiconductors. Nat. Energy.

[80] Roß M, Severin S, Stutz MB, Wagner P, Köbler H, Favin-Lévêque M, Al-Ashouri A, Korb P, Tockhorn P, Abate A, Stannowski B, Rech B, Albrecht S (2021). Co-evaporated formamidinium lead iodide based perovskites with 1000 h constant stability for fully textured monolithic perovskite/silicon tandem solar cells. Adv. Energy Mater..

[81] Bett AJ, Schulze PSC, Winkler KM, Kabakli ÃS, Ketterer I, Mundt LE, Reichmuth SK, Siefer G, Cojocaru L, Tutsch L, Bivour M, Hermle M, Glunz SW, Goldschmidt JC (2020). Two-terminal perovskite silicon tandem solar cells with a high-bandgap perovskite absorber enabling voltages over 1.8 V. Prog. Photovoltaics.

[82] Luo X, Luo H, Li H, Xia R, Zheng X, Huang Z, Liu Z, Gao H, Zhang X, Li S, Feng Z, Chen Y, Tan H (2023). Efficient perovskite/silicon tandem solar cells on industrially compatible textured silicon. Adv. Mater..

